# Recent Understandings of Pet Allergies

**DOI:** 10.12688/f1000research.7044.1

**Published:** 2016-01-27

**Authors:** Dennis Ownby, Christine Cole Johnson

**Affiliations:** 1 Georgia Regents University, Augusta, GA, USA; 2 Department of Public Health Sciences, Henry Ford Health System, Detroit, MI, USA

**Keywords:** Pet Allergies, Allergic reactions, IgE antibodies, allergic sensitization

## Abstract

Allergic reactions to pets have been recognized for at least a hundred years. Yet our understanding of the effects of all of the interactions between pet exposures and human immune responses continues to grow. Allergists, epidemiologists, and immunologists have spent years trying to better understand how exposures to pet allergens lead to allergic sensitization (the production of allergen-specific immunoglobulin class E [IgE] antibodies) and subsequent allergic disease. A major new development in this understanding is the recognition that pet exposures consist of not only allergen exposures but also changes in microbial exposures. Exposures to certain pet-associated microbes, especially in the neonatal period, appear to be able to dramatically alter how a child’s immune system develops and this in turn reduces the risk of allergic sensitization and disease. An exciting challenge in the next few years will be to see whether these changes can be developed into a realistic preventative strategy with the expectation of significantly reducing allergic disease, especially asthma.

## Introduction

Many families in the US and other countries keep a variety of pets. The American Pet Products Association estimates that 65% of US households own a pet, an increase of 3% from 2010 to 2015. Although this estimate may be high, there is little doubt that approximately half of all American families have a pet and the vast majority of these pets are furry ^1, 2^. It is also well known that when there is a high prevalence of pet-keeping in a community, pet allergens are found in relatively high concentrations in public places such as schools. These “second-hand” exposures to pet allergens have been shown to exacerbate disease in sensitive children ^3^. The purpose of this review is to provide an understanding of some of the recent studies related to pet allergies and potential health consequences. The areas to be explored include the following: the changing prevalence of all types of allergic diseases, how people’s interactions with pets have changed, new concepts of how the human immune system responds to pet allergens, and especially the growing understanding of how this immune response relates to the microbial ecology of the gut. The potential economic costs of pet allergies will also be briefly explored. There are many excellent reviews focusing on the precise mechanisms of human immune responses to pets ^4– 9^.

This review will focus on scientific research related to human allergic responses to pets. However, it is important to recognize that pet allergy can be a very emotionally sensitive topic. Every practicing allergist has repeatedly heard a family say that “if ‘X’ person in the family is allergic to the pet, then ‘X’ goes and the pet stays in the home!”. This statement is always meant to be humorous but it clearly comes from strong feelings about the pet’s place in the family. This is but one example of how pets are commonly considered members of the family and considerable angst is generated if there is a conflict between the potential health of a family member and the love of the pet. The point to be made is that strong emotions may lead to strongly held beliefs that have little factual basis. The internet has widely spread information on topics such as “low” or “hypoallergenic” pets when there is little supporting scientific evidence ^10, 11^.

This review will focus on cats and dogs since these are the most popular pets in the US and many other developed countries. Because cats and dogs are the most prevalent household pets, there are many more studies of how they relate to allergic disease. Although reactions to other animals will not be examined in detail, allergic responses to other animals are believed to be similar to those elicited by cats and dogs after considering the relative intensity and duration of exposures, and many studies have shown that the majority of pet allergens come from the same protein family ^12, 13^. An important cross-over area is occupational exposure versus in-home exposure to animals. For example, in both settings, exposure to mice or rats can be desired (pets or reared animals) or unwanted (vermin). The literature in these areas is voluminous but beyond the scope of this review.

An important topic that is beyond this review is the relationship between exposure to cats and dogs and the risk of allergic disease. This subject has been widely debated and subjected to numerous reviews but will not be included herein ^4, 14– 19^.

## Temporal increase in prevalence of allergies

Many studies over the previous two to three decades have suggested that allergic diseases have increased in frequency ^20^. Most notable has been the increase in asthma over the past three decades. Although some would argue that there have been epidemics of different allergic diseases at different times, it is difficult to discern how many of these changes were related to gradually improving recognition and diagnosis of new diseases ^20^. However, there is little doubt that asthma has nearly tripled in prevalence among youth in the US since the 1970s and that food allergies have at least doubled in the same peroid ^21, 22^. The reasons for the increase in the prevalence of allergies have been widely debated and investigated but there is no consensus on the precise cause. Among the many hypotheses are improving hygiene, global warming, increasing use of antibiotics (especially in food), and reduced physical activity. One of the few agreed-upon assumptions is that the increase has occurred during one or two generations, making genetic evolution highly improbable; however, a new area of inquiry is epigenetic change, which is the heritable change in gene expression that does not involve changes to the underlying DNA sequence. There are at least three mechanisms of epigenetic change: DNA methylation, histone modification, and non-coding RNA-associated gene silencing ^23^. Epigenetic changes have been linked to asthma in some studies ^24– 27^.

## Changing relationships of people to pets

Objective data concerning how persons view their pets and the role of pets in families over multiple generations are extremely hard to find. A study by Kennedy and McGarvey took an interesting approach to examining this question of whether the relationship between pets and their families has changed over time ^28^. They examined 1,348 advertisements including both people and pets which had appeared in popular US women’s magazines from the 1920s through the 1980s and coded the ads for seven themes such as whether the pet was depicted indoors or outdoors, was on a leash, or was used to depict companionship (i.e. touching or holding the pet). They concluded that over the time interval studied, pets have moved from outdoor protectors to indoor family members ^28^.

The increasingly close contact between persons and pets and the resulting higher allergen exposures occurred over the same decades during which all allergic diseases appeared to be increasing. This combination has been thought to be a major reason for the increase in pet allergies. Unfortunately, data supporting this hypothesis are not robust. In the US, skin test results from the NHANES (National Health and Nutrition Examination Survey) II and III studies were compared. NHANES II was conducted from 1976 through 1980, and NHANES III was from 1988 through 1994. There were six allergens common to both studies, allowing comparison of results in a representative sample of the non-institutionalized, US population, from ages 6 to 59 years. The probability of reacting to at least one allergen skin test was higher in NHANES III compared with II: 41.9% (standard error [SE] 1.23) and 21.8% (SE 0.94), respectively, slightly more than a doubling. Reactivity to cat, the only pet allergen tested in both NHANES II and III, increased 5.5-fold (3.1% to 17.0%) compared with an average increase of 2.6-fold for the other five in common allergens (rye and Bermuda grasses, short ragweed, oak, and *Alternaria alternate*). Tempering this finding is the possibility that the cat allergen used in NHANES III was more potent, thus increasing reactivity; however, studies from Europe suggest similar changes ^29^.

## Immune response to pet allergens

Allergy has always been defined by the presence of immunoglobulin class E (IgE) antibodies immunologically specific for individual antigens. Initially, the minute quantities of IgE present in humans could be detected only by allergen skin testing. Now laboratory tests for cat allergens are essentially as sensitive as skin tests, but the results of skin and *in vitro* tests are not always identical ^29, 30^. Antigens eliciting IgE responses are referred to as allergens. Although IgE antibodies have been most extensively studied, humans do produce other immune responses to allergens. Immune responses are initiated by specialized antigen-presenting cells such as dendritic cells, which present the allergen to T cells. Attempts have been made to identify the small portions of the major cat allergen ( *Felix domesticus* 1, abbreviated Fel d 1) presented to T cells, in the hope of using these peptides to induce hyporesponsiveness to Fel d 1 as a treatment for cat allergy ^31, 32^. Recent studies have identified how the cysteine-rich portion of the major cat allergen, Fel d 1, is bound on cells through a mannose receptor ^33^. Some have suggested that Fel d 1 is uniquely able to induce an IgG subclass 4 (IgG4) response in many individuals and that high concentrations of Fel d 1–specific IgG4 can block IgE responses ^34, 35^. However, other studies have not found a relationship between cat-specific IgE and IgG4 levels and symptoms ^36, 37^. Multiple studies, including those of allergen immunotherapy with cat and other allergens, all suggest that repeated relatively high-dose exposure to any allergen leads to IgG4 production ^7, 38, 39^.

Interestingly, the allergens characterized from furry animals thus far have all belonged to three broad groups of proteins: secretoglobins, lipocalins, and kallikreins. Whereas Fel d 1 is a secretoglobin of unknown function, more than 50% of allergens from furry animals have been identified as lipocalins ^12, 13^. These animal allergens are found in dander, saliva, and urine. They are commonly on small particles that allow airborne dispersion and also dispersion by adherence to surfaces such as clothing ^2, 40^. The apparent constant circulation of pet allergens on shoes and clothing through public areas and into homes has made it very difficult to control symptoms from pet allergens by avoidance measures such as air cleaning ^41, 42^.

## Microbial exposures related to pets and other animals

Probably the most dramatic change in understanding the relationships between pet exposure and pet allergy is the realization that pet exposure involves more than just exposure to the allergens shed by the pet ^43^. Multiple studies have shown that early life exposure to pets and to farm animals is associated with a reduced risk of subsequent allergic disease ^44– 46^. Although other studies have disputed these findings, the results of systematic reviews and meta-analyses have typically shown either a reduction or no increase in risk associated with infantile exposure to furry pets ^47– 50^. The hypothesis developed to explain why animal exposure could be associated with a decreased prevalence of allergy postulates that animals increase the diversity of microbes to which a child is exposed, and that this more diverse exposure leads to the development of an immune system less likely to develop allergic responses to antigens. Two studies have demonstrated that cats or dogs in the home increase the diversity of the microbiota of the home ^43, 51^. Another study showed that the stool microbiota of children living with pets differed from those without pet exposure. Studies in homes of farmers also suggest a broader diversity of microbes ^52– 54^. Several investigations have been directed toward understanding the dominant exposures of farm living leading to a lower prevalence of allergic disease. One important factor in farm living is consumption of farm (i.e. unpasteurized) milk ^55– 57^. The assumption is that farm milk contains many live bacteria that can alter the gut microbiota of the child, or that unpasteurized milk contains substances supporting the growth of specific microbes. This hypothesis is supported by studies showing that the amount of bacterial contamination in surface water used for drinking is directly correlated with a lower risk of allergic disease ^58^. Variables that appear relatively consistent in all of these studies are (1) that the exposure to diverse bacteria must occur in during the first year of life and perhaps in the first weeks of life, and (2) that the types of bacteria which appear to be protective are common soil bacteria or bacteria found in the gastrointestinal tracts of mammals. This increasing knowledge related to microbial exposure has led some to suggest that we shift from the “hygiene” to the “microbial” hypothesis of allergen protection ^59, 60^. The critical question is whether this knowledge can be developed into a medically valuable preventive strategy such as supplementing mothers or infants with live bacteria at a critical stage of development.

Although the full demonstration of this animal-microbe-gut-immune development hypothesis has not been achieved, multiple human and animal studies strongly support the hypothesis. Several early studies have shown that there are differences in stool microbes between children in certain countries and that these changes are associated with the risk of asthma ^61^. One of the most supportive mouse studies was by Fujimura *et al.* ^62^. These investigators first compared gavaging young adult mice with slurries of house dust from homes with and without dogs. The mice were then immunized with cockroach allergen by using a protocol designed to induce allergic sensitivity and asthma-like airway reactivity. The mice given the dust from homes with a dog were strongly protected from sensitization and airway disease compared with mice given the house dust from homes without dogs. An analogous experiment using challenge with respiratory syncytial virus (RSV) again showed strong protection of the mice given dust from homes with dogs. When the microbial communities of the caeca of the gavaged mice were examined, several different microbes were in much higher abundance in the mice given dust from homes with dogs. One of these bacteria, *Lactobacillus johnsonii*, was cultured and given to groups of mice. The mice given gavages with live *L. johnsonii* were again significantly protected from both allergen-induced and RSV infection-induced airway disease, but gavages with killed bacteria were not effective. It has been shown that supplementing high-risk infants with *Lactobacillus casei* subsp. Rhamnosus (LGG) does alter the development of the gut microbiome and so it may be possible to use supplementation as a disease prevention strategy in humans ^63^. Others have shown similar protective effects from *Lactobacillus reuteri* in mice ^64^. There are also suggestions that vitamins and other diet elements may play roles in altering the gut microbiome and the subsequent function of the immune system ^65, 66^.

While the hypothesis of infants acquiring a different gut microbiome from animal exposure has been developing, there are other findings that suggest this hypothesis is missing essential elements. An alternative hypothesis is that pregnant women living with pets or closely associated with farm animals may over time develop different gut and vaginal microbiomes and that these pet-associated maternal gut microbiomes are inoculated into infants during normal vaginal birth. This would fit with the studies showing that birth by C-section carries a higher risk of childhood asthma than vaginal birth, presumably because an infant does not acquire a large inoculum of maternal vaginal and gut microbes at birth ^67– 69^. Another study related to this hypothesis is the finding that the prenatal presence of dogs in homes has a stronger effect on the development of total serum IgE in infants delivered by C-section than those born vaginally ^70^. It could be argued that if pets were associated with an alteration of the maternal gut or vaginal microbiome, a stronger effect would have been expected in infants born vaginally. An alternative argument is that infants born by C-section have a suboptimal initial maternal inoculum, which allows a greater impact by environmental microbes such as those in house dust on early colonization ^68, 71, 72^.

A common argument against the hygiene and microbial hypotheses related to allergy is the frequently quoted high prevalence of asthma among inner-city residents ^73– 75^. However, a recent study from the Inner-City Asthma Consortium found a clear interrelationship between allergen exposure, microbe exposure, and risk of disease ^18^. In that study, children with the greatest exposure to allergens and bacteria in specifically the first year of life had the lowest risk of recurrent wheeze and allergic sensitization, again suggesting the protective effect from exposure to a high diversity of bacteria. Unfortunately, that study was racially homogenous and so potential effects of race could not be evaluated.

An important element to consider in all studies of pets and of microbes is the timing of exposure related to immune development. As already suggested, the effects of pets on the development of the infant gut microbiome are likely to be much larger in the first weeks and months of life than in later childhood. The one-year age cutoff found in many studies may be an artifact of how data were collected rather than a biological horizon.

A common question related to the apparent protective effect of early pet exposure on allergy is whether this is of clinical significance and if so how the information should be used. The current level of understanding is inadequate at best. If our hypothesis that early pet exposure may alter an infant’s developing microbiota and lead to a reduced risk of some immune diseases is correct, then the critical question is whether this knowledge can be transformed into a therapeutic strategy. Clearly, exposing all children to pets is not possible and probably not desirable. There are many questions related to owning a pet to consider: costs of food, veterinary care, possible zoonotic infections, etc. These are questions that persons should carefully consider before obtaining a pet. However, if a pregnant woman has a pet when she finds that she is pregnant, we believe that there is no increased risk of allergic disease and probably a decreased risk if she continues to keep the pet through the birth of her child and the child’s first year. After the first year of life, the data become inconsistent. Some studies suggest that continued pet exposure after the first year of life provides additional protection from atopy whereas other studies do not find any benefit after the first year ^76– 78^. The more important question is whether knowledge of the interaction of pet and human microbiota can be used to provide a preventive option such as a probiotic supplement. Although such trials have been conducted with mixed results, it appears that much more study and understanding are necessary before there will be consistent success with such approaches ^79, 80^. Other potentially simple approaches to allergy reduction that may be related to microbial exposures, such as hand washing of dishes and licking pacifiers, seem to be helpful with minimal risks ^81, 82^.

## Health-care costs related to pet allergy

The potential allergy-related health-care costs of keeping pets are rarely discussed in the medical literature, partially because these costs are difficult to objectively assess. One question is whether pet-keeping by pet-allergic individuals with asthma substantially increases costs of asthma care. A study estimated that the increased number of visits for acute asthma care among dog-allergic adults, who chose to live with dogs, might add as much as $0.5 to 1.0 billion per year to costs of care in 2010 ^83^. This estimate suggests a substantial increase in health-care cost for adults but does not include indirect costs such a lost work days which would drive the estimate even higher ^83^. Unfortunately, there have not been any similar estimates of how pet allergy might increase the cost of asthma care for children. However, Almqvist *et al.* showed that even indirect exposure to cat dander, brought into classrooms on the clothes of children living with cats, increased symptoms and medication use in cat-allergic children with asthma ^84^. The increased costs of new-onset asthma in a child are also difficult to estimate but substantial. A longitudinal study of 3,535 school children in California identified only three risk factors for new-onset asthma in these children: a humidifier (relative risk [RR] 1.7, confidence interval [CI] 1.2–2.4), any pet (RR 1.6, CI 1.0–2.5), and having a dog in the home (RR 1.4, CI 1.0–2.0) ^85^. Similarly, in other studies, sensitization to cats or dogs has been identified as a risk factor for new-onset reactive airway disease ^86, 87^. The presence of a dog in the home also increased ozone exposure-related asthma symptoms in a study ^88^. In total, these studies suggest that allergic sensitivity to pets and pet exposure are significant contributors to the overall costs of asthma care.

The combination of the studies summarized in this review shows a somewhat paradoxical relationship of pets and allergy. Exposure to the microbes associated with pets in the first few months of life appears to be associated with a substantial reduction in the risk of allergic disease and asthma. This effect appears to last at least until early adulthood ^78^. Only one longitudinal study has shown that continuing exposure to dogs was required for continuing protection at least until 7 years of age ^76^. Others have not shown any apparent effect after the first year ^77^. A few studies have shown that sensitization to cats or dogs is a risk factor for new-onset asthma later in life, and one study has shown that the presence of a dog in the home was a risk factor for new-onset asthma.

## Summary

Pets are an important source of health benefits to many individuals. But close contact with pets, such as when they live in homes, can be associated with a variety of risks, including medically significant allergic diseases. Adding confusion to our understanding of the relationship of pets to allergic disease has been the discovery that infantile exposure to furry pets appears to be associated with a substantial reduction of allergy and asthma risks in childhood; however, it is possible that continuing pet exposure may become a risk for allergies and asthma at some stage of life. The apparent allergy-protective effect of pets appears to be mediated through exposure to a more diverse microbial community in the home. The discovery of this microbe-related protective effect will hopefully lead to allergy prophylactic options in the coming years without requiring direct pet or other animal exposure.
